# Acute and sub-chronic toxicity of *Cajanus cajan* leaf extracts

**DOI:** 10.1080/13880209.2017.1309556

**Published:** 2017-05-11

**Authors:** Rong Tang, Ru-hua Tian, Jia-zhong Cai, Jun-hui Wu, Xiao-ling Shen, Ying-jie Hu

**Affiliations:** a Tropical Medicine Institute, Guangzhou University of Chinese Medicine, Baiyun Qu, Guangzhou, China;; b Pi-Wei Institute, Guangzhou University of Chinese Medicine, Baiyun Qu, Guangzhou, China

**Keywords:** Maximal tolerated doses, organ weight, stilbene, flavonoid, phytoestrogen, HPLC

## Abstract

**Context:** The leaves of *Cajanus cajan* (L.) Millsp. (Fabaceae) have diverse bioactivities, but little safety data are reported.

**Objective:** This study examines the toxicological profiles of *C. cajan* leaf extracts.

**Materials and methods:** The leaves were extracted by water or 90% ethanol to obtain water or ethanol extract (WEC or EEC). EEC was suspended in water and successively fractionated into dichloroform and *n*-butanol extracts (DEC and BEC). Marker compounds of the extracts were monitored by high-performance liquid chromatography (HPLC). Kunming mice were administered with a single maximum acceptable oral dose (15.0 g/kg for WEC, EEC and BEC and 11.3 g/kg for DEC) to determine death rate or maximal tolerated doses (MTDs). In sub-chronic toxicity investigation, Sprague–Dawley rats were orally given WEC or EEC at 1.5, 3.0 or 6.0 g/kg doses for four weeks and observed for two weeks after dosing to determine toxicological symptoms, histopathology, biochemistry and haematology.

**Results:** Flavonoids and stilbenes in the extracts were assayed. In acute toxicity test, no mortality and noted alterations in weight and behavioural abnormality were observed, and the maximum oral doses were estimated as MTDs. In sub-chronic toxicity study, no mortality and significant variances in haematological and biochemical parameters or organ histopathology were observed, but increased kidney weight in 3.0 g/kg WEC- or 3.0 and 6.0 g/kg EEC-treated female rats, and reduced testes and epididymis weight in EEC-treated male rats were recorded. These changes returned to the level of control after recovery period.

**Conclusion:** Acute and sub-chronic toxicity of *Cajanus cajan* leaf extracts was not observed.

## Introduction

*Cajanus cajan* (L.) Millsp. (Fabaceae) is a perennial shrub distributed in the tropics and subtropics of Asia, Africa and the Americas, and known as pigeon pea. In China, *C. cajan* leaves are used as a folk medicine to relieve swelling and pain, kill parasites and treat varicella (Guangdong Food and Drug Administration 2004; Nanjing University of Chinese Medicine 2012). More than 40 chemical constituents have been identified from *C. cajan*, and flavonoids and stilbenoids are chief bioactive constituents (Bhanumati et al. 1978; Cooksey et al. 1982; Chen et al. 1985; Zhang et al. 2012; Li et al. 2014). The extracts of *C. cajan* leaves are reported to have bioactivities such as antimalarial (Duker-Eshun et al. 2004), cytotoxic (Ashidi et al. 2010), hypolipidemic (Luo et al. 2008; Ye et al. 2013), bone loss reduction (Zheng et al. 2007), enhancement of osteogenesis (Zhang et al. 2010; Cai et al. 2015) and bone density (Ye et al. 2013). An extract of *C. cajan* leaf with therapeutic effect on aseptic necrosis of the femoral head has been developed as a pharmaceutical (Yuan et al. 2005; China Food and Drug Administration 2007). Recently, anti-inflammatory, antinociceptive, immunomodulatory and antioxidant activities of *Cajanus cajan* seeds cultivated in Egypt were also reported (Hassan et al. 2016). However, little information in toxicity and safety evaluation on pharmacologically active extracts from *C. cajan* is reported. Here, we address this deficit by describing data from animal models treated with *C. cajan* leaf extracts.

## Materials and methods

### Plant collection and identification

The leaves of *C. cajan* were collected from Yuanmou County, Yunnan Province of China in October 2013 and authenticated by Professor Fu-Wu Xing at South China Botanical Garden, Chinese Academy of Sciences. A voucher specimen (No.SD201310) was deposited at Tropical Medicine Institute, Guangzhou University of Chinese Medicine.

### Preparation of the water extract of CCL (WEC)

Air-dried *C. cajan* leaves (10.00 kg) were extracted two times with 15-fold boiling water (150 kg) under reflux (1 h × 2). The aqueous extract was concentrated *in vacuo* to 10 L (a volume (L) equals to the weight (kg) of leaves), cooled, mixed with 95% ethanol to form a mixture containing 70% ethanol and filtrated. The filtrate was concentrated and dried *in vacuo* to yield WEC (1.31 kg; 13.1%).

### Preparation of the ethanol extract of CCL (EEC), the dichloromethane fraction (DEC) and the n-butanol fraction (BEC) of EEC

The air-dried leaves of *C. cajan* (15.00 kg) were extracted three times with 15-fold 90% ethanol (230 L) under reflux (1 h × 3). The combined extractive solution was concentrated and dried under reduced pressure to yield EEC (1.38 kg; 9.23%). EEC was suspended in water and extracted with dichloromethane. The dichloromethane solution was concentrated and dried *in vacuo* to afford DEC (600 g, 3.99%). The remaining water phase was extracted with *n*-butanol likewise and yielded BEC (261 g, 1.74%).

All of the extracts were stored at −20 °C and dissolved (WEC and BEC) or dispersed (EEC and DEC) in 0.5% sodium carboxymethyl cellulose (Na-CMC; vehicle) before use.

### Experiment animals

Kunming (KM) mice (20–30 days-of-age, 16–20 g; with certificate No. SCXK (YUE) 2008-002) and Sprague Dawley (SD) rats (30–40 days-of-age, 100–140 g; with certificate No. SCXK (YUE) 2013-002) from Guangdong Center of Experimental Animal (Foshan, Guangdong, China) were used in acute and sub-chronic toxicity investigations. Animals were housed in stainless steel cages (*N* = 5 rats/mice), and males and females were housed separately at a room temperature of 25 ± 1 °C and a relative humidity of 60% ± 5% with a 12-h light–dark cycle. Animals had *ad libitum* access to food and water immediately and during the acclimatization period (two days for mice and five days for rats) before experiment. During the acute toxicity test, mice (*N* = 50 male; 50 female) were randomly divided into four experimental groups and a control group (WEC, EEC, DEC, BEC and control; *N* = 10/sex/group). For sub-chronic toxicity tests, rats (*N* = 70 male; 70 female) were randomly assigned to six treatment groups and a control group (WEC or EEC, each treated with three doses, and control, treated with vehicle; *N* = 10/sex/group). All animal experiments were approved by the Animal Ethics Committee of Guangzhou University of Chinese Medicine (Protocol No. S20140001) and followed the Animal Care and Use Guidelines.

### Acute oral toxicity

The study was in accordance with the acute toxicity study rudder of Chinese traditional medicine, natural medicine (China Food and Drug Administration 2005a). Four treatment groups were randomly assigned to receive a single and maximal orally administered dosage of the extracts (WEC, EEC or BEC at 15.0 g/kg, and DEC at 11.3 g/kg). Control animals received vehicle. After fasting for 8 h, mice were orally administered with extract or equivalent volume of vehicle (20 mL/kg) and allowed free access to food and water 3 h after dosing. Animal weight, behaviour, toxicity and mortality were monitored continuously for 5 h immediately after dosing and then observed daily for 14 days. Mice were sacrificed and hearts, livers, spleens, lungs, kidneys and stomachs were removed and assessed.

### Sub-chronic oral toxicity

The sub-chronic oral toxicity was assessed according to the long-term toxicity study ‘rudder’ of Chinese traditional medicine, natural medicine (China Food and Drug Administration 2005b). WEC and EEC doses were set as 1.5, 3.0 and 6.0 g/kg. All rats had *ad lib* access to food and water and were orally dosed with 10 mL/kg WEC or EEC suspension once daily (treatment groups) or equivalent volume of vehicle (control). Mortality was monitored daily, and animal weight, behaviour and toxicity were recorded twice a week over 28 days and a 14-day recovery period.

### Pathology

Half of the experimental rats in each group (5 females; 5 males) were sacrificed after the exposure period via 10% chloral hydrate (ip), and gross necropsy was performed. Organs (heart, liver, spleen, lung, kidney, ovary, uterus, testis and epididymis) were recorded and expressed as organ weight. For paired organs, the combined weight of both organs was used. Tissues of organs were fixed with 10% neutral buffered formalin, processed and stained with haematoxylin and eosin for histopathological examination. Haematological and serum biochemical data were also measured. The remaining animals were fed for another two weeks without treating with extracts. At the end of the recovery period, animals were treated the same as these animals depicted here.

### Blood sampling

Blood was collected from the abdominal aorta and placed in tubes with or without EDTA for later measurements of erythrocytes, leucocytes, haemoglobin, haematocrit, mean corpuscular volume and haemoglobin, and platelet count with an animal special automatic blood analyser (Sysmex XT-2000iv, Sysmex, Japan). Samples collected in non-anticoagulant vials were centrifuged at 3000 rpm for 10 min to obtain serum, and ALT, AST, total protein, albumin, alkaline phosphatase activity (AP), total bilirubin (TBIL), urea and creatinine were measured with an automatic biochemistry analyser (7020, Hitachi, Japan).

### Statistical analysis

Data are expressed as means ± SD. Data variance in data for animal and organ weight and haematological and serum biochemistries were assessed for homogeneity with Levene's procedure. If the variance was homogeneous, the data were assessed by one-way analysis of variance followed by the Dunnett *post hoc* test. If not, the Tamhane T2 test was applied. Differences at 95% confidence levels (*p* < 0.05) were considered significant. Statistical analyses were performed using SPSS 17.0 statistical analysis software (SPSS Inc., Chicago, IL).

### HPLC assay

Flavonoid glycosides orientin, vitexin and genistin; stilbenes longistyline A and longistyline C; and dihydroxyflavone pinostrobin, which were isolated from *C. cajan* leaves and identified by us, were used as markers for high-performance liquid chromatographic (HPLC) assay on the *C. cajan* leaf extracts. With an Ecosil-C_18_ (4.6 mm ×250 mm, 5 μm) column, separation of orientin, vitexin and genistin in WEC and BEC was well achieved by gradient elution with methanol (A) and 0.1% phosphoric acid (B) as mobile phase: 0 min (28% A) → 60 min (28% A) → 80 min (50% A) at oven temperature 40 °C (detection wavelength 268 nm; flow rate 0.8 mL/min), while qualitative and quantitative measurements of pinostrobin, longistyline A and longistyline C in EEC and DEC were achieved by gradient elution with methanol (A) and water (B): 0 min (70% A) →10 min (80% A) → 25 min (85% A) → 35 min (100% A) at 30 °C (detection wavelength 290 nm; flow rate 1.0 mL/min).

## Results

### Acute toxicity

**Figure 1.**
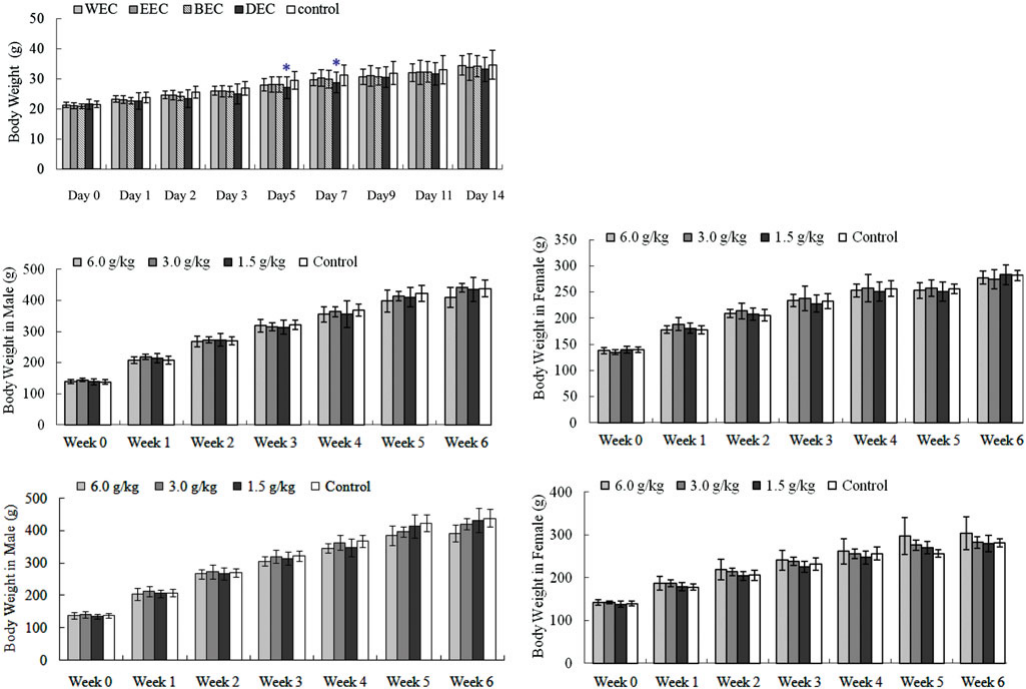
Effects of oral *Cajanus cajan* leaf extracts on body weight (g). (A) Kunming mice treated with WEC, EEC, BEC or DEC in acute toxicity test. Data are means ± SD (*N* = 20; half male and half female). (B) Male Sprague Dawley (SD) rat treated with WEC in sub-chronic toxicity, (C) female SD rat treated with WEC in sub-chronic toxicity, (D) male SD rat treated with EEC in sub-chronic toxicity, (E) female SD rat treated with EEC in sub-chronic toxicity. Data are means ± SD (*N* = 10 rats/group/sex in 4-week treatment period; *N* = 5 rats/group/sex for 2-week recovery period). *p < 0.05 vs. control.

No animals died and behaviours such as grooming, respiration, reflex and animal weight after treatments were unchanged. Animal weight gains after WEC, EEC and BEC treatment were consistent with controls (61–64%) and ∼10% greater than animals treated with DEC (Figure 1(A) andTable 1). DEC-treated animals on days 5 and 7 had a transient decrease in body weight (*p <* 0.05 vs. control group) (Figure 1(A)), and no other alterations were noted in all groups. Accordingly, MTDs of WEC, EEC, DEC and BEC were estimated to be 15.0, 15.0, 11.3 or 15.0 g/kg, respectively.

**Table 1.** Animal weight and relative weight gain (RWG) of Kunming mice before and after administration with the maximal oral dose of *Cajanus cajan* leaf extract or vehicle: acute toxicity assessment.

Day/RWG	Control	WEC	EEC	BEC	DEC
0	21.53 ± 1.12	21.35 ± 0.86	21.10 ± 0.97	20.89 ± 0.85	21.58 ± 1.64
14	34.69 ± 4.79	34.52 ± 3.32	33.93 ± 4.45	34.21 ± 3.49	33.20 ± 4.03
RWG	61%	62%	61%	64%	54%

Data are expressed as means ± SD (*N* = 20 animals, half male and half female). RWG: Relative weight gain = (Mean weight gain at day 14/Mean weight at day 0) × 100%.

### Sub-chronic oral toxicity

#### Behavioural observation

All rats survived throughout the 4-week treatment period. Compared to controls, reduced motility, abdominal distention and poor grooming in high-dose (6.0 g/kg) EEC-treated rats were observed.

#### Body weight

Animal weight gained of WEC (1.5, 3.0 or 6.0 g/kg), EEC (1.5 or 3.0 g/kg) treatment and control were consistent and positive after the 4-week exposure period and again during the 2-week recovery period (Table 2). Male rats treated with high dose (6.0 g/kg) of EEC lost ∼15% of their body weights (*p <* 0.05 vs. control) at the end of recovery period, while in all EEC-treated groups of female rats, the weight gain was accordant (Table 2 and Figure 1).

**Table 2.** Animal weight and relative weight gain (RWG) of SD rats treated with WEC, EEC or vehicle after a 4-week treatment period and a 2-week recovery period: sub-acute toxicity.

			WEC			EEC		
Sex	Week/RWG	Control	1.5 g/kg	3.0 g/kg	6.0 g/kg	1.5 g/kg	3.0 g/kg	6.0 g/kg
Male	0	137.92 ± 7.80	139.00 ± 9.71	143.99 ± 5.18	139.41 ± 6.70	135.27 ± 7.72	141.02 ± 10.49	138.24 ± 9.33
	4	368.48 ± 18.71	355.13 ± 42.65	363.44 ± 16.60	354.87 ± 24.87	347.15 ± 26.67	362.29 ± 23.31	346.04 ± 15.45
	RWG	167%	155%	152%	155%	157%	157%	150%
	6	438.18 ± 27.37	435.10 ± 38.20	440.79 ± 13.78	409.25 ± 33.01	431.72 ± 36.53	419.96 ± 18.14	391.99 ± 25.69*
	RWG	218%	213%	206%	194%	219%	198%	184%
Female	0	139.53 ± 4.96	139.34 ± 6.61	134.77 ± 4.83	137.66 ± 5.45	137.80 ± 6.79	142.10 ± 2.73	142.66 ± 6.34
	4	256.33 ± 15.20	250.66 ± 17.93	257.53 ± 26.55	253.01 ± 12.50	247.54 ± 15.26	255.53 ± 11.11	261.65 ± 29.54
	RWG	84%	80%	91%	84%	80%	80%	83%
	6	281.49 ± 11.14	283.12 ± 21.22	274.38 ± 19.96	277.49 ± 13.58	297.56 ± 43.22	282.26 ± 14.05	304.07 ± 38.71
	RWG	102%	103%	104%	102%	116%	99%	113%

Values are expressed as means ± SD (*N* = 20; 10 rats/sex/group during 4-week exposure period; *N* = 5 rats/sex/group during 2-week recovery period). Relative weight gain = (Mean weight at the end of exposure or recovery/Mean weight at week 0) × 100%. *p < 0.05 vs. control.

#### Organ weight

**Figure 2.**
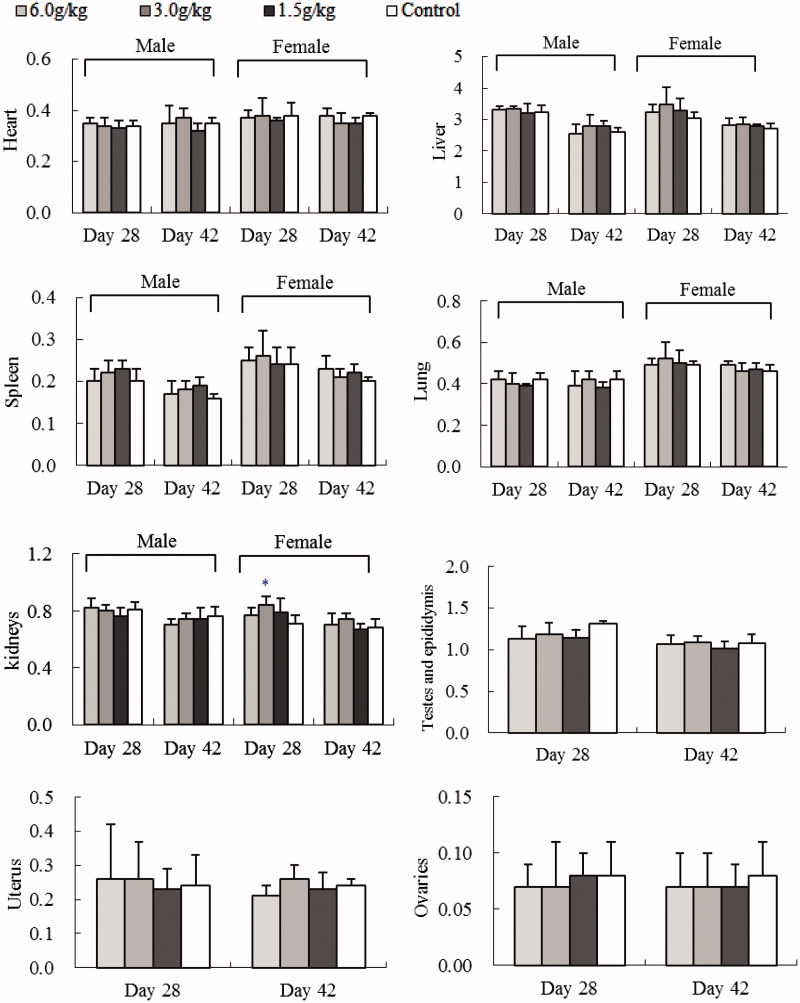
Effect of oral WEC on relative SD rat organ weight (g): sub-chronic toxicity. Data are means ± SD (*N* = 10; 5 rats/group/sex for 4-week treatment and 2-week recovery period), *p < 0.05 vs. control.

**Figure 3.**
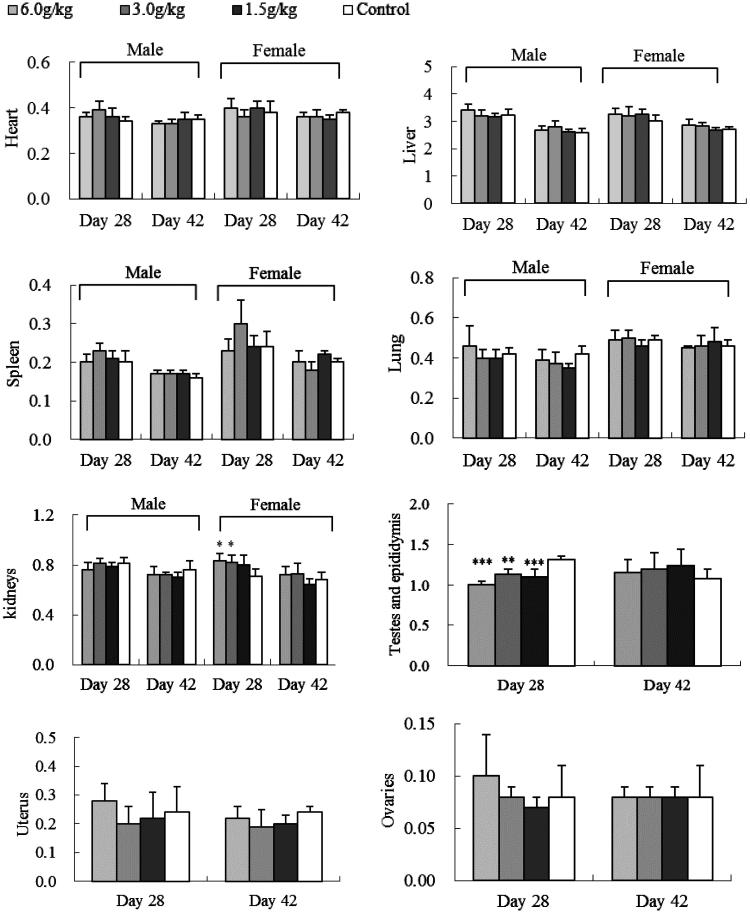
Effect of oral EEC on relative SD rat organ weight (g): sub-chronic toxicity. Data are means ± SD (*N* = 10; 5 rats/group/sex for 4-week treatment and 2-week recovery period), *p < 0.05, ** *p <* 0.01, ***p < 0.001 vs. control.

After the 4-week exposure of water or ethanol extracts of *C. cajan* leaves, change of animal organ weights was different. Female rat kidneys were greater after WEC (3.0/kg)- and EEC (3.0 or 6.0 g/kg) treatments (*p <* 0.05 vs. control) (Figures 2 and 3). Male rats had smaller testes and epididymis at all EEC doses (*p <* 0.001 or *p <* 0.01 vs. control) (Figure 3). After a 2-week recovery period, these organ weight differences were not significant (Figures 2 and 3).

#### Haematology and serum biochemistry

Haematological or biochemical differences in any animal after any treatment at any time were not different (*p* > 0.05 vs. control) (Figures S1–S4, Supporting Information).

#### Histopathology

There was no marked organ change noted at necropsy of the rats in treatment groups or control group in the exposure and recovery periods (Figure S5, Supporting Information).

#### Compositional HPLC assay of extracts

**Figure 4.**
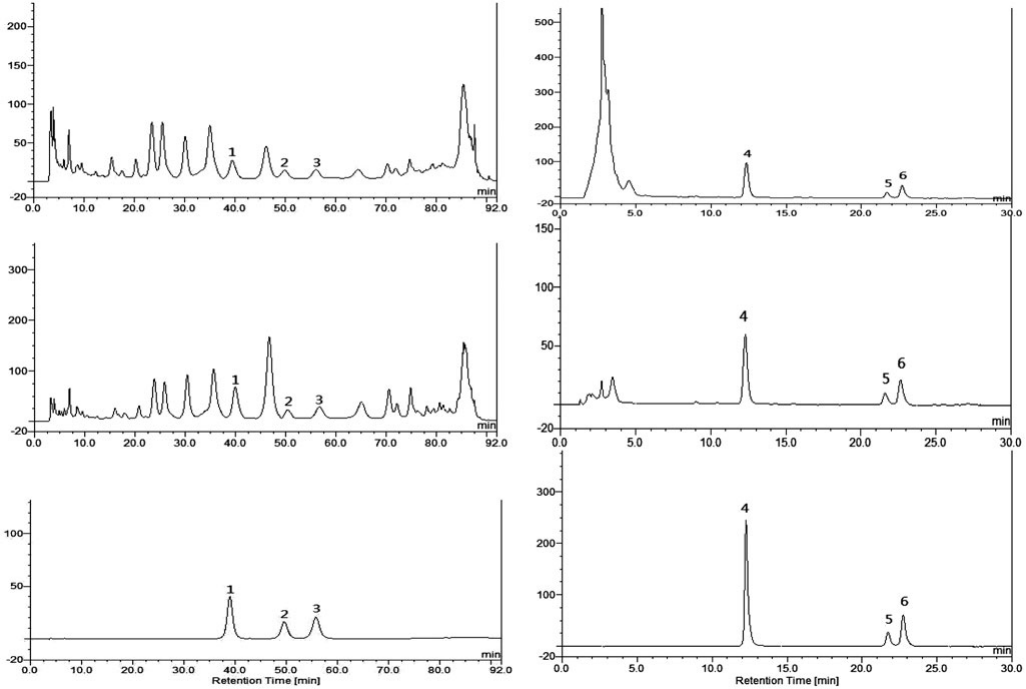
HPLC chromatograms of *Cajanus cajan* leaf extracts and reference compounds. (A) WEC, (B) BEC, (D) EEC, (E) DEC, (C) mixed water-soluble reference compounds, (F) mixed lipophilic reference compounds.1. Orientin, 2. Vitexin, 3. Genistin, 4. Pinostrobin, 5. Longistyline A, 6. Longistyline C.

Figure 4 and Table 3 depicted HPLC analytical results of *C. cajan* leaf extracts. HPLC data showed that components of WEC and BEC were flavonoid glycosides, and DEC contained weak polar stilbenoids and flavonoid genin(s). Orientin, vitexin and genistin were 0.25%–0.78% in WEC and 0.51%–2.98% in BEC; contents of pinostrobin, longistyline A and longistyline C were 0.22%–1.06% in EEC and 1.80%–7.34% in DEC.

**Table 3.** HPLC assay for flavonoid and stilbenoid marker compounds in *Cajanus cajan* leaf extract (mg/g).

Extract	Orientin	Vitexin	Genistin	Extract	Pinostrobin	Longistyline A	Longistyline C
WEC	7.78 ± 0.02	2.68 ± 0.02	2.47 ± 0.02	EEC	10.55 ± 0.11	2.18 ± 0.04	4.27 ± 0.06
BEC	29.78 ± 0.17	5.11 ± 0.05	7.28 ± 0.04	DEC	73.40 ± 0.59	18.00 ± 0.09	32.35 ± 0.25

Values are expressed as mean ± SD from three independent experiments.

## Discussion

Plants have long been used to treat human disease, and many plants have turned into the basis of medicines. Chemical constituents and medicinal purposes of *C. cajan* leaves have been studied for over 30 years; however, little toxicological data on *C. cajan* leaves are reported. Here, we presented the MTD for four *C. cajan* leaf extracts (15.0 g/kg for WEC, EEC and BEC, and 11.3 g/kg for DEC), which signified no acute toxicity at these doses in KM mice. Oral administration of WEC or EEC for 4 weeks at three doses did not appear to alter SD rat behaviour, weight, feeding, haematology or biochemistry, or cause pathological organ lesions. Thus, these doses (1.5–6.0 g/kg) were safe.

Animal weight changes are critical for toxicological study. In acute toxicity test, animal weight gains after treatment suggested that the MTDs may not be toxic to the degree that feeding diminished and weight was lost in KM mice. SD rats had the same outcomes in sub-chronic toxicity test. One transient weight decrease (*p <* 0.05 vs. control) in mice treated with DEC and a significant decrease (*p <* 0.05 vs. control) in male rats treated with 6.0 g/kg EEC were noted (Figure 1 and Table 2). These outcomes were ascribed to decreased nutrient absorption and assimilation due to gastric disturbances due to treatments because we noted abdominal distention and reduced motility. Thus, our data suggest that these extracts were likely not toxic to these animals at the doses given and the serum data supported this assessment. For variances in haematological and biochemical data among all treatment groups were not different from control (Figures S1–S5, Supporting Information).

The major constituents in *C. cajan* leaves are phytoestrogenic isoflavones including genistein, 2′-hydroxylgenistein, cajanol and cajanin, and stilbenes such as longistyline A, longistyline C, cajaninstilbene acid and 1-carboxylic-2,4-dimethoxy-3-prenyl-stilbene (Cooksey et al. 1982; Dahiya et al. 1984; Duker-Eshun et al. 2004). In our investigation, phytoestrogens of isoflavones and stilbenes were detected in the ethanol and water extracts, EEC and WEC, by HPLC (Table 3 and Figure 4). Phytoestrogens may adversely affect the development and function of male reproductive organs in the long term (from conception to adulthood) via exposure to high-soya bean-contained dietary (Lee et al. 2004; Cederroth et al. 2010). In the sub-chronic toxicity study, female rats exposed to 3.0 or 6.0 g/kg EEC, or 3.0 g/kg WEC for 4 weeks had increased kidney weight (*p* < 0.05 vs. control) and male rats exposed to all doses of EEC for 4 weeks had smaller testes and epididymis weight (*p* < 0.01 and *p* < 0.001 vs. control) (Figures 2 and 3). These differences disappeared after a 2-week recovery period, and serum data did not change (Figures S1–S5, Supporting Information). Thus, we tentatively speculate the renal and testicular/epididymis weight change is phytoestrogen-connected. West et al. (2005) reported that the adverse effects of dietary oestrogens on male reproductive function can be reversible. Likewise, affects from EEC and WEC on organ weight in female rat kidney and testes and epididymis were normalized after the exposure of extracts discontinued.

## Conclusions

Overall, the acute and sub-chronic oral toxicity of the extracts from *C. cajan* leaves was not observed at the test doses. Interestingly, treated for 4 weeks, WEC or EEC increased the kidney weight in female SD rats, while EEC decreased the testes and epididymis weight in male SD rats. These affected organ weights, accompanying unvaried haematological, biochemical and histopathological assessments in the same period, restored to the level of control after recovery period, are considered as the influences of phytoestrogens from the extracts.

## Supplementary Material

Yingjie_Hu__et_al_supplemental_content.zip
