# Supplementary material for: Acute and sub-chronic toxicity of Cajanus cajan leaf extracts
Source: Pharm Biol. 2017 May 11;55(1):1740–6. doi: 10.1080/13880209.2017.1309556 (PMC6130582; doi:10.1080/13880209.2017.1309556)
Supplement: Yingjie_Hu__et_al_supplemental_content.zip [file IPHB_A_1309556_SM2357.zip › Yingjie Hu et al supplemental content.doc]

A	B

C	D

E	F

G	H

**Figure S1.** Hematology of Sprague Dawley (SD) rats treated with WEC: in sub-chronic toxicity. A. Leucocytes (WBC), B. erythrocytes (RBC), C. hemoglobin (HGB), D. hematocrit (HCT), E. mean corpuscular volume (MCV), F. mean corpuscular hemoglobin (MCH), G. mean corpuscular hemoglobin concentration (MCHC), H. platelet count (PLT). Data are means ± SD (N= 10; 5 rats/group/sex, at 4-week exposure period and 2-week recovery period).

A	B

C	D

E	F

G	H

**Figure S2.** Hematology for SD rats treated with EEC: sub-chronic toxicity. A. Leucocytes (WBC), B. erythrocytes (RBC), C. hemoglobin (HGB), D. hematocrit (HCT), E. mean corpuscular volume (MCV), F. mean corpuscular hemoglobin (MCH), G. mean corpuscular hemoglobin concentration (MCHC), H. platelet count (PLT). Data are means ± SD (N= 10; 5 rats/group/sex, at 4-week exposure period and 2-week recovery period).

A	B

C	D

E	F

G	H

**Figure S3.** Serum biochemistries of SD rats treated with WEC: sub-chronic toxicity. A. ALT, B. AST, C. total protein (TP), D. albumin (ALB), E. total bilirubin (TBIL), F. alkaline phosphatase activity (AP), G. urea, and H. creatinine (CRE). Data are means ± SD (N= 10; 5 rats/group/sex, at 4-week exposure period and 2-week recovery period).

A	B

C	D

E	F

G	H

**Figure S4.** Serum biochemistries of SD rats treated with EEC: sub-chronic toxicity. A. ALT, B. AST, C. total protein (TP), D. albumin (ALB), E. total bilirubin (TBIL), F. alkaline phosphatase activity (AP), G. urea, and H. creatinine (CREA). Data are means ± SD (N= 10; 5 rats/group/sex, at 4-week exposure period and 2-week recovery period).

A	B	C

Heart

Liver

Spleen

Lung

Kidneys

Stomach

Colon

Uterus

Testes

Epididymis

**Figure S5.** Photomicrograph of H&E histology of tissues of SD rats after *C. cajan* leaf extract treatment: sub-chronic toxicity. No lesions were observed at necropsy for any rat in any treatment group or in any control. A, control group; B, WEC 6.0 g/kg; C, EEC 6.0 g/kg. Magnification 200 ×.
